# Presence of Shiga Toxin-Producing *Escherichia coli* (STEC) in Fresh Beef Marketed in 13 Regions of ITALY (2017)

**DOI:** 10.3390/microorganisms6040126

**Published:** 2018-12-06

**Authors:** Bianca Maria Varcasia, Francesco Tomassetti, Laura De Santis, Fabiola Di Giamberardino, Sarah Lovari, Stefano Bilei, Paola De Santis

**Affiliations:** Istituto Zooprofilattico Sperimentale Lazio e Toscana, “M. Aleandri”, 00178 Rome, Italy; biancamaria.varcasia@izslt.it (B.M.V.); francesco.tomassetti@izslt.it (F.T.); laura.desantis@izslt.it (L.D.S.); fabiola.digiamberardino@izslt.it (F.D.G.); sarah.lovari@izslt.it (S.L.); stefano.bilei@izslt.it (S.B.)

**Keywords:** *Escherichia coli* (STEC), beef, serogroups, *stx*-genes, *stx*-subtypes

## Abstract

The aim of this study was to determine the prevalence of Shiga toxin-producing *Escherichia coli* in fresh beef marketed in 2017 in 13 regions of Italy, to evaluate the potential risk to human health. According to the ISO/TS 13136:2012 standard, 239 samples were analysed and nine were STEC positive, from which 20 strains were isolated. The STEC-positive samples were obtained from Calabria (*n* = 1), Campania (*n* = 1), Lazio (*n* = 2), Liguria (*n* = 1), Lombardia (*n* = 1) and Veneto (*n* = 3). All STEC strains were analysed for serogroups O26, O45, O55, O91, O103, O104, O111, O113, O121, O128, O145, O146 and O157, using Real-Time PCR. Three serogroups were identified amongst the 20 strains: O91 (*n* = 5), O113 (*n* = 2), and O157 (*n* = 1); the O-group for each of the 12 remaining STEC strains was not identified. Six *stx* subtypes were detected: *stx1a*, *stx1c*, *stx2a*, *stx2b*, *stx2c* and *stx2d*. Subtype *stx2c* was the most common, followed by *stx2d* and *stx2b*. Subtype *stx2a* was identified in only one *eae*-negative strain and occurred in combination with *stx1a*, *stx1c* and *stx2b*. The presence in meat of STEC strains being potentially harmful to human health shows the importance, during harvest, of implementing additional measures to reduce contamination risk.

## 1. Introduction

Shiga toxins (Stx) are potent cytotoxins encoded by lambdoid phages and integrated into the bacterial chromosome of a large and complex group of pathogenic *Escherichia coli* (STEC) strains that cause disease in humans [1,2]. Shiga toxins are immunologically distinct [3] and based on this antigenic diversity are divided into two groups, Stx1 and Stx2 [4]. Epidemiological studies [3,4,5] have shown that some Stx1 and Stx2 subtypes often are associated with severe human STEC illnesses [4]. Three Stx1 variants have been identified: Stx1a, Stx1c and Stx1d [2,4]. Usually, subtypes Stx1c and Stx1d are found in the meat of sheep, deer, and wildlife [6,7,8,9] and are rarely associated with disease in humans [2]. Subtypes Stx2a, Stx2b, Stx2c, Stx2d, Stx2e, Stx2f and Stx2g, along with the recently discovered Stx2h, are the eight Stx2 subtypes known to exist [4,10,11,12,13,14]. Subtypes Stx2a, Stx2c and Stx2d are associated with STEC infections in humans [11,15,16,17], while Stx2e, Stx2f, Stx2g are mainly found in animals [2,10,11]. Some studies have suggested that STEC strains producing Stx2f can cause diarrhoea in humans [18]; however, recently, STEC strains carrying the *stx2f* gene have been isolated from patients with hemolytic uremic syndrome (HUS) [18,19].

The majority of STEC strains associated with disease in humans possess adherence factors that facilitate their attachment to the intestinal epithelial cells [20]. The principal adherence factor is the intimin protein encoded by the *eae* gene, and responsible for what is known as the “attaching and effacing” (A/E) lesion of the intestinal mucosa [4,21,22,23]. The simultaneous presence of *eae* and *stx2* genes is considered a reliable indicator of a particular STEC strain’s ability to cause severe disease in humans [24]. However, STEC strains that lack the *eae* gene can also cause severe disease by utilising alternative adherence mechanisms, as evidenced recently during a large outbreak of HUS in Germany in 2011 and caused by an enteroaggregative haemorrhagic *Escherichia coli* (EAHEC) O104:H4 carrying the *aggR* and *aaiC* genes in combination with *stx2a* [25].

Serological identification, based on the somatic (O) and flagellar (H) antigens, has to date resulted in the identification of ~470 STEC serotypes [8,26], all able to produce any one of the twelve known Stx subtypes or combinations of these subtypes [27]. The European Food and Safety Authority (EFSA) has identified STEC encoding the *stx* and *eae* genes that belong to serogroups O26, O103, O111, O145, O157, the so-called “big five”, as those of major concern to human health in Europe [28]. Following the O104:H4 outbreak in Germany, this serotype was incorporated into the screening protocol for all *eae*-negative STEC isolated from food (Regulation EU No. 209/2013) [29].

STEC O157 is the most frequently reported serogroup worldwide [17,30,31]. The incidence of STEC O157 has however decreased in recent years, whereas the so-called non-O157 STEC serogroups are increasingly associated with haemorrhagic colitis (HC) and HUS in humans [27,31]; the most frequently encountered non-O157 serogroups are O26, O103, O111, O121, O145, O45, O118, O71 and O186 [31,32]. In 2015, as reported by EFSA and the European Centre for Disease Prevention (ECDC), in Europe, the STEC serogroups most commonly isolated from beef were O157 and O26, followed by O148, O145, O8, O113, O91, O130, O174 and O113. Many of these STEC serogroups were linked to human illnesses, confirming the epidemiological involvement of beef in STEC infections [33]. In 2016, in Europe, the STEC serogroup most frequently isolated from bovine meat was O157, followed by O113, O26, O145 and O174 [34]. In Italy, STEC O26 was the predominant serogroup in 2012 and responsible for about half of STEC cases in humans, followed by STEC O157 and STEC O111 [35]. In Europe, as a consequence of only a handful of countries doing any monitoring, few data exist on the isolation of STEC from beef [1,21,34].

The main reservoirs of STEC are ruminants, including wildlife. STEC can colonize the gut asymptomatically, their excretion into the environment [21] serving as a significant route of infection in humans [3]. Other studies have demonstrated that the hides of cattle represent an important source of STEC, resulting in carcass contamination during harvest [21,36]. Transmission to cattle may take place on-farm or during transportation to the abattoir [36,5]. STEC prevalence in cattle appears to be influenced by the age of the animal, the season, and probably, also feed composition [17,5]. Pathways along which humans may become infected include faecal-oral contamination during harvest, direct contact with faeces, STEC cross-contamination and multiplication during the preparation and handling of animal-derived foodstuffs, and human-to-human transmission [21,37]. European legislation (Regulation EU No. 2073/2005 and its amendments Regulation EU No. 1441/2007) [38,39] did not include the screening of STEC from meat products because, originally, very few data were available on the health risks associated with STEC-contaminated food [1,21].

In 2012, the International Organisation for Standardisation (ISO) issued the ISO/TS 13136:2012 method for the detection of STEC with a focus on the *stx1* and *stx2* virulence genes and on the *eae* adhesion factor gene, as these are associated with the “big five” serogroups [40]. The method is based on the Real-Time PCR screening of enrichment cultures, followed by serogroup identification and characterisation of isolated strains. The initial enrichment step, by increasing concentrations of the target bacteria, not only enhances the sensitivity of the method but also ensures the viability of bacterial cells from which positive results are obtained [41]. To date, as stated in the ESFA and ECDC 2017 report on trends and sources of zoonoses, zoonotic agents and food-borne outbreaks, 91.5% of the samples tested during 2016 by the European Member States, were analysed using ISO/TS 13136:2012 [34]. Some studies have suggested that the culture conditions involving media formulations and incubation temperature, as currently recommended in the ISO, be modified to further enhance STEC growth [41,42,43,44]. While improvements to the current ISO standard are possible, food authorities will always promote the use of a standardized method so results from different countries remain comparable [34].

The aim of this study was to determine the prevalence of Shiga toxin-producing *Escherichia coli* in fresh beef marketed in 13 regions of Italy in 2017, to evaluate the potential risk to human health.

## 2. Materials and Methods

### 2.1. Sampling

Between January and December 2017, 239 samples of refrigerated fresh beef were obtained from the retail market in 13 regions of Italy. The samples were collected originally to monitor antimicrobial resistance in zoonotic bacteria from food-producing animals and meat, under Decision 2013/652/EC [45]. The antimicrobial resistance aspects do not form part of this study, but they provided us with the opportunity to assay samples that were representative of most of Italy. The 13 regions account for >90% of the total animals harvested in Italy. The samples were arbitrarily chosen from supermarkets and traditional butcheries, and were obtained at least once monthly throughout the year to cover all four seasons. A single sample was collected from each lot of origin, either domestic or imported; frozen meat was excluded. The samples included meat either sliced or diced, vacuum-wrapped, or packaged under a controlled atmosphere. The 239 samples were obtained from the regions of Abruzzo (*n* = 8), Calabria (*n* = 7), Campania (*n* = 3), Emilia Romagna (*n* = 23), Friuli Venezia Giulia (*n* = 6), Lazio (*n* = 34), Liguria (*n* = 6), Lombardia (*n* = 53), Marche (*n* = 7), Piemonte (*n* = 25), Puglia (*n* = 22), Toscana (*n* = 20) and Veneto (*n* = 25).

### 2.2. Screening of Enrichment Cultures

The samples were analysed following the ISO/TS 13136:2012 standard [40]. Twenty-five grams of meat homogenised with 225 mL of modified Tryptone Soya Broth (mTSB) (Biolife Italiana srl, Milan, Italy) supplemented with 16 mg/mL of novobiocin (Biolife Italiana srl, Milan, Italy) and incubated at 37 °C for 18–24 h. DNA was extracted from 1 mL of each enrichment culture, using an automated nucleic acid purification system (MagPurix^®^ 12S, Resnova, Rome, Italy), following the manufacturer’s instructions. The extraction method provided approximately 100 ng/μL of DNA eluted in nuclease-free water at a final volume of 200 μL. DNA extracts were tested for the *stx1*, *stx2* and *eae* genes by Real-Time PCR, following the ISO standard procedure given above. PCR amplifications were done maintaining a final volume of 20 μL that contained 3 μL of DNA template (standardized at a concentration of 20 ng/μL), 1× qPCR Master Mix (Kapa Biosystems, Resnova, Rome, Italy), 300 nM of each primer, and 125 nM of each probe (Eurofins Genomics, Milan, Italy). All the reactions included an internal amplification control (Exo IPC kit) (Eurogentec, Italy). PCR conditions comprised an enzyme activation step of 95 °C for 5 min, followed by 40 cycles: 95 °C for 3 s (denaturation) and 60 °C for 30 s (annealing/extension/data acquisition). All the reference material used as reaction positive controls were provided by the European Union Reference Laboratory for *E. coli* (EU-RL VTEC). All *stx*-positive and *eae*-positive enrichment broths were screened for serogroups O26, O111, O103, O145 and O157 [40], while the *stx-*positive but *eae*-negative broths were screened also for the O104 serogroup following an additional protocol provided by the EURL VTEC [46]. All the serogroup Real-Time PCRs were done using the same reagent formulas and PCR conditions described above for the *stx* and *eae* genes; only for serogroup O103 was the annealing/extension temperature lowered to 55 °C.

### 2.3. Isolation of STEC Strains

For STEC strain isolation, the *stx*-positive enrichment broths were cultured on Tryptone Bile X-Glucuronide (TBX) agar (Biolife Italiana srl, Milan, Italy) or, if screening of the enrichment broths indicated the presence of serogroup O26, were cultured also on Rhamnose MacConkey (RMAC) agar and incubated at 37 °C for 18–24 h. Then, of many colonies that phenotypically resembled *E. coli*, fifty were selected arbitrarily and re-analysed singly for the presence (or absence) of the *stx* and *eae* genes using Real-Time PCR. Based on the original enrichment broth results, STEC colonies were tested for one or more of the “big five” serogroups. Those *stx*-positive colonies that tested negative for the “big five”, were then analysed for the O45, O55, O91, O113, O121, O128 and O146 serogroups, using a method provided by the EURL VTEC [46].

### 2.4. Stx Subtyping

The *stx* subtype of each STEC strain was identified using the PCR-based subtyping protocol of the Statens Serum Institut, WHO Collaborating Centre for Reference and Research on *Escherichia* and *Klebsiella* [11,9]. The PCR was done using the 2GFast Master mix (Resnova, Rome, Italy), 280 nM of each primer (Eurofins, Milan, Italy) and 5 μL of template DNA (20 ng/μL). Each reaction was adjusted to a final volume of 25 μL in nuclease-free water. The annealing temperature was 66 °C for subtyping *stx1a-c*, 62 °C for *stx2a-c*, and 64 °C for *stx2d-g*. Agarose gel electrophoresis was used to visualize the PCR products. A molecular weight marker (Euroclone S.p.a., Milan, Italy) was used to assign the molecular weight to amplicons produced. The samples were run in Tris-Borate-EDTA running buffer (VWR International Srl, Milan, Italy) at a constant voltage (100 V for the first ten minutes and 60 V until the end of electrophoresis). All Statens Serum Institut reference material used as reaction positive controls was provided through the EURL VTEC. 

## 3. Results

### 3.1. Real-Time PCR Screening of Enrichment Cultures, and Isolation of STEC Strains

During initial Real-Time PCR screening of enrichment cultures, *stx* genes were detected in 20 (8.4%) of the 239 samples. STEC was not isolated from 11 of these 20 *stx*-positive enrichment cultures, hence, based on the ISO/TS 13136:2012, in these samples only the “presumptive” presence of STEC could be determined. The *eae* gene was detected in eight of the eleven “presumptive” enrichment cultures, *stx1* in seven, and *stx2* in ten. One or more of the “big five” serogroups occurred singly, or in combination, in five enrichment cultures, as follows: O104 (*n* = 1), O103 (*n* = 1), O104 + O111 (*n* = 1), O26 + O103 + O157 (*n* = 2). For the six remaining “presumptive” STEC positive cultures, the serogroup was not identified. The regions of Italy from which “presumptive” positive STEC samples were obtained, are provided along with the sampling month in Table 1.

Twenty STEC strains were isolated from nine of the 20 *stx*-positive enrichment cultures, hence the samples are classified as STEC “presence”. The STEC-positive samples were obtained from the regions of Calabria (*eae* + *stx1* + *stx2*; *n* = 1), Campania (*stx2*; *n* = 1), Lazio (*eae* + *stx1* + *stx2* and *stx1* + *stx2*; *n* = 2), Liguria (*stx1* + *stx2*; *n* = 1), Lombardia (*stx2*; *n* = 1) and Veneto (*eae* + *stx1* + *stx2*; *n* = 3). Three different serogroups were identified amongst the 20 strains isolated: O91 (*n* = 5), O113 (*n* = 2), and O157 (*n* = 1). The remaining 12 STEC strains tested negative for all the serogroups analysed (O26, O45, O55, O91, O103, O104, O111, O113, O121, O128, O145, O146 and O157). 

### 3.2. Stx Subtyping

Subtyping of the *stx* genes detected in the 20 STEC isolates, displayed various *stx1* and *stx2* subtype profiles. Strains belonging to serogroup O91 (*n* = 5) carried five different virulence gene profiles (*eae* + *stx1a*; *eae* + *stx1c*; *stx2c*; *eae* + *stx1a* + *stx1c* + *stx2c*; *eae* + *stx1a* + *stx1c* + *stx2c* + *stx2d*). Two O113 isolates were *eae*-negative and contained subtypes *stx2c* and *stx2c* + *stx2d*, respectively. One O157 strain was *eae*-positive and comprised *stx* subtypes *stx1a* + *stx2c* + *stx2d*. Twelve STEC isolates tested negative to all 13 serogroups analysed (nd, Table 2); amongst these isolates various combinations of *eae* and *stx* subtypes were detected, with *stx2c* found most frequently, followed by *stx2d*, *stx2b* and *stx2a*, respectively. Finally, subtypes *stx1d*, *stx2e*, *stx2f* and *stx2g* were not detected in any of the 20 STEC isolates obtained (Table 2).

### 3.3. Discussion

Cattle are considered a major reservoir for virulent strains of Shiga toxin-producing *Esherichia coli* (STEC) and the most important source of human infections through the consumption of contaminated beef products. The aim of this study was to identify and characterise the STEC strains found to occur in fresh beef obtained from 13 regions in Italy. A culture method involving selective and non-selective media, and following an initial enrichment step, was used to isolate STEC strains [1,47]. Specific PCR assays were used to identify pathogenicity factors (*eae* and *stx* genes), serogroups, and *stx* subtypes [1,17,40,48,49]. Initial enrichment yielded 20 (8.4%) *stx*-positive cultures, while STEC strains were only isolated from nine cultures. The failure to isolate STEC from a *stx*-positive enrichment culture has been reported upon previously [21,50,51,52]. To isolate STEC from food can be challenging because the number of STEC cells are likely to be low; other hurdles include sublethal cell injury, or cell growth suppressed in the presence of a large population of competing microflora [4,21,53]. For these reasons, enrichment cultures are essential to augment sensitivity, thereby promoting the isolation of STEC strains needed to confirm the presence of the *stx* genes in the live cell, while excluding the presence of free DNA or free prophages in the cultures [28]. Recently, various authors have reported the reduced sensitivity of mTSB enrichment broths supplemented with novobiocin (16 mg/L), suggesting that a decrease in novobiocin concentration might improve detection of O111 and other non-O157 serogroups [41,42,43,44]. While it is possible that reduced concentrations of novobiocin facilitate the isolation of non-O157 serogroups, 19 of the 20 isolates obtained represented non-O157 STEC strains. 

In this study, overall STEC contamination in beef was 3.8%, a prevalence rate that agrees only partly with rates obtained previously in Italy. A frequency rate of 0.42% for STEC O157 matches that obtained during a nationwide survey conducted by Conedera et al. [54] and who reported STEC in four (0.43%) of 931 minced beef samples. These were screened only for serogroup O157. In the region of Piemonte, Rantsiou et al. [55] found six (5.9%) STEC strains in 101 mixed meat products using a method developed in-house. In the Emilia Romagna region, Bardasi et al. [56], following the ISO/TS 13136:2012 protocol, demonstrated an STEC presence in four (0.6%) of 689 meat samples (representing pork, bovine and poultry). In a more recent study, the same ISO protocol was used to test 675 pork samples (comprising both fresh and dried products) collected in the Umbria and Marche regions of Italy [57]; these authors reported the presumptive presence of *stx*-genes in 2.8% of the products, but were unable to isolate any STEC strains. The discrepant STEC prevalence rates obtained may find causes in various factors, including geographic compartmentalization of the *E. coli* population amongst food animals, laboratory techniques and protocols employed [54,55], and the wide range in meat products analysed. According to EFSA, in Europe, the overall presence of STEC in 18,975 food samples assayed was 2.5%, the highest proportion found in meat, particularly that from small ruminants [34]. In Switzerland, Fantelli et al. reported the presence of STEC in 2.3% of 211 minced beef samples tested [58]; in France, 4% of 411 beef samples were STEC positive [59]. Our STEC prevalence rates are comparable to some of those obtained for beef previously in Europe [34,58,59].

Real-Time PCR, based on the O-antigen synthesis genes (*wzx* and *wzy*), is widely used to serogroup STEC strains [60]. However, the Real-Time PCR methods currently used do not cover all known serogroups, hence many serogroups to which a STEC strain may belong remain unidentified [33]; consequently, 12 of our 20 STEC strains could not be identified to serogroup. While serogroups and serotypes are not virulence factors and not predictive of a virulence profile, they nevertheless remain useful for conducting surveillance and for investigating outbreaks [61]. Serogroups O26, O103, O104, O111 and O157, along with the *eae* and *stx2* virulence genes, were detected in 11 enrichment broths; the failure to isolate STEC strains from these 11 broths is the reason why the corresponding samples were classified as “presumptive” positive. The potential risk to human health that “presumptives” represent, means the responsible authorities must continue to monitor for STEC. 

An association between Stx subtype and severity of disease in humans has been observed [51,62]. In this study, 20 STEC strains were isolated from nine beef samples and carried *stx* subtypes in various combinations. The *stx1* subtypes detected were *stx1a* and *stx1c*. The Stx1a toxin subtype is often produced by strains that are *eae*-positive and known to cause severe disease in man [32]. In this study, nine STEC strains, either *eae*-positive or *eae*-negative, had *stx1a* alone or in combination with *stx1c* or *stx2a*, *stx2b*, *stx2c* and *stx2d*. The Stx1c toxin subtype is reported mainly in *eae*-negative strains causing mild infections [63]; we found *stx1c* both in identified (serogroup O91) and unidentified serogroups that were *eae*-positive (Table 2). With regard to toxin type Stx2, the subtypes *stx2a*, *stx2c* and *stx2d* have been linked to HC and HUS in humans [11,16]. In this study STEC isolates carrying *stx2c* were the *stx* subtypes most commonly found, followed by *stx2d* and *stx2b*. Subtype *stx2a* was identified in only one *eae*-negative strain and occurred in combination with *stx1a*, *stx1c* and *stx2b*. Of the eight *eae*-positive STEC strains obtained, four belonged to serogroup O91, one to O157, while three represented unidentified serogroups. Two strains of O113 and one of O91 were *eae*-negative; the nine remaining strains were not identified to serogroup (Table 2). Contrary to other reports [64], we found four O91 STEC strains to be *eae*-positive; this finding is unusual and we cannot explain it satisfactorily. Subtype *stx2d* was found in eight STEC strains, of which four were *eae*-negative, while the other four were *eae*-positive; the latter group included a strain O157 that was also *stx1a*- and *stx2c*-positive. The *stx2d* subtype is usually associated with *eae*-negative strains and severe disease in humans [65]; recently, in Spain, Sanchez et al. [66] reported upon a O157:H7 strain that was *eae*- and *stx2d*-positive and isolated from a 2-year-old child with BD. This unusual virulence combination, though rare, has been reported also from several HUS-affected patients in France and separately involving STEC O26:H11 [67] and STEC O80:H2 [68].

## 4. Conclusions

In Italy, the isolation of STEC strains from fresh meat samples signals the recurring threat that beef, consumed either undercooked or raw, poses to human health. The variety of *stx* types and subtypes and multiple STEC serogroups detected, are amongst those found elsewhere in the world and where, in humans, they have been demonstrated to be involved in severe diseases, such as BD, HC and HUS. The presence in meat of potentially harmful STEC strains emphasizes the importance, during harvest, of implementing additional measures to reduce contamination risk. Linked to this, an efficient surveillance strategy for STECs in retail foodstuffs, remains a national priority. The laboratory diagnostic protocols needed to isolate and accurately identify STEC strains are laborious, expensive, and time-consuming. However, they continue to remain pivotal to assessing the strain of pathogenic *E. coli* involved, and for identifying the possible source of infection. This knowledge is needed to enable the competent authorities to respond precisely and rapidly. Improvements to current isolation techniques, and the validation and standardization of molecular protocols, remain a matter of urgency. It is foreseen that in the future new high-power methodologies, such as Next-Generation Sequencing (NGS), will become more widely utilised and that these will lead to further improvements in the currently used standards for diagnosing STEC in foods. 

## Figures and Tables

**Table 1:** STEC “presumptive” presence. Intimin (*eae*), Shiga-toxin *stx1* and *stx2* genes and serogroups detected in enrichment broth cultures obtained from fresh beef samples collected within 13 regions of Italy (2017).

Region of Italy	STEC Presumptive Presence (No)	Sampling Month	Sample ID	STEC Virulence Gene Profile			*E. coli* Serogroup
				*eae*	*stx1*	*stx2*	
Abruzzo	1	May	42696	+	+	+	nd ^1^
Lazio	2	September	78963		+	+	nd ^1^
		October	82856			+	O104
Liguria	1	June	51045	+	+	+	O103
Lombardia	4	August	64370			+	nd ^1^
		September	72350	+		+	O104-O111
		September	75247	+	+	+	O26-O103-O157
		November	96150	+	+	+	nd ^1^
Marche	1	November	97189	+		+	nd ^1^
Puglia	1	July	57025	+	+		nd ^1^
Veneto	1	October	87734	+	+	+	O26-O103-O157

^1^ nd (not determined): enrichment broth cultures that tested negative to the “big five” serogroups analysed (O26, O103, O111, O145, O157) and O104.

**Table 2:** Subtyping of Shiga-toxin stx1 and stx2 genes and serogroups associated with STEC strains isolated from fresh beef samples collected within 13 regions of Italy (2017).

Region of Italy	Samples Positive (No)	Sampling Month	Strain ID	*stx* Subtypes ^1^						*eae*	Serogroup
				*stx1a*	*stx1c*	*stx2a*	*stx2b*	*stx2c*	*stx2d*		
Calabria	1	November	94200-01	+						+	O91
			94200-02		+					+	O91
			94200-03	+	+			+	+	+	nd ^2^
			94200-04	+	+			+	+	+	O91
Campania	1	December	107402-01					+			nd ^2^
Lazio	2	June	50231-01					+			O91
			50231-02	+				+	+	+	O157
			50231-03					+			O113
		November	107400-01					+	+		O113
			107400-02		+		+				nd ^2^
			107400-03	+				+	+		nd ^2^
			107400-04					+	+		nd ^2^
Liguria	1	December	104076-01	+	+	+	+				nd ^2^
Lombardia	1	July	58927-01				+	+			nd ^2^
Veneto	3	March	27847-01				+				nd ^2^
		March	22774-01					+	+		nd ^2^
			22774-02					+			nd ^2^
		November	94197-01	+	+			+		+	O91
			94197-02	+	+			+		+	nd ^2^
			94197-03	+	+			+	+	+	nd ^2^

^1^*stx* subtypes included *stx1a-d* and *stx2a-g*; subtypes *stx1d*, *stx2e*, *stx2f* and *stx2g*, were not detected in any of the isolated STEC strains. ^2^ nd (not detemined): strains that tested negative to all serogroups analysed (O26, O45, O55, O91, O103, O104, O111, O113, O121, O128, O145, O146 and O157).
